# Hydroxycinnamoyl Amino Acids Conjugates: A Chiral Pool to Distinguish Commercially Exploited *Coffea* spp.

**DOI:** 10.3390/molecules25071704

**Published:** 2020-04-08

**Authors:** Federico Berti, Luciano Navarini, Silvia Colomban, Cristina Forzato

**Affiliations:** 1 Dipartimento di Scienze Chimiche e Farmaceutiche, Università degli Studi di Trieste, via L. Giorgieri 1, 34127 Trieste, Italy; fberti@units.it; 2 Illycaffè S.p.A., via Flavia 110, 34147 Trieste, Italy; Luciano.Navarini@illy.com (L.N.); Silvia.Colomban@illy.com (S.C.)

**Keywords:** hydroxycinnamoyl amides, coffee beans, LC-MS

## Abstract

The synthesis of five hydroxycinnamoyl amides (HCAs) was accomplished and their identification and quantification in the green coffee bean samples of *Coffea* *arabica*, *Coffea* *canephora*, and *Coffea* *liberica* was performed. The HCAs *p*-coumaroyl-*N*-tyrosine **1b**, caffeoyl-*N*-phenylalanine **2b**, caffeoyl-*N*-tyrosine **3b**, and *p*-coumaroyl-*N*-tryptophan **4b** were characteristic of the *C. canephora* species while caffeoyl-*N*-tryptophan **5b** was present in both *C. canephora* and *C. arabica*, but with higher content in *C. canephora*. The HCAs presence was also analyzed in *C. liberica* for the first time and none of the targeted compounds was found, indicating that this species is very similar to *C. arabica* species. Between *C. canephora* samples from various origins, significant differences were observed regarding the presence of all the HCAs, with *C. canephora* from Tanzania containing all five derivatives.

## 1. Introduction

Hydroxycinnamic acid conjugates are secondary metabolites widely present in the plant kingdom, such as endive (*Cichorium endivia*) [1], purple coneflower (*Echinacea purpurea*) [2], African star grass (*Hypoxis hemerocallidea*) [3], lady fern (*Athyrium filix-femina*) [4], Robusta coffee (*Coffea canephora*) [5], and cocoa (*Theobroma cacao*) [6]. Hydroxycinnamic acids can be esterified with quinic acid forming the class of chlorogenic acids, esterified with monosaccharides forming the cinnamoyl glycosides, or can give origin to amides with different amino acids, named hydroxycinnamoyl amides (HCAs) or *N*-phenylpropenoyl-amino acids. The hydroxycinnamoyl acids are usually conjugated with the amino acids phenylalanine, tyrosine, tryptophan, or aspartic acid, and although their role in the plant is not known, they exhibit biological activity, such as anti-neuroinflammatory activity [7], hepatoprotective activity [8], HIV-1 integrase inhibitors [9], and antidiabetic effects [10]. Moreover, from a sensory point of view, HCAs have been indicated as possible contributors to the sensory properties of some food [11].

More than 124 species are known of the genus *Coffea* and the two most important ones are *Coffea arabica* L., simply known as Arabica, and *Coffea canephora* Pierre ex Froehner, simply known as Robusta, which correspond to 57% and 43% of the international coffee trade, respectively, according to the International Coffee Organization (ICO, 2019). *Coffea liberica* Bull ex Hiern, contributing less than one per cent of the marketed coffee, is the third commercially exploited coffee species, well known for its larger cherries, when compared with those of *C. arabica* or *C. canephora*. *C. liberica* is less cultivated, due to its lower caffeine content (1.8% dry matter basis), and to its sensitivity to *Fusarium xyloriodes*, despite the fact that this species was of great economic importance during the 1930–1950 period [12]. Due to its low economic importance, *C. liberica* is also the less studied of the three considered species.

The HCAs can contribute to distinguish the two mainly commercially exploited coffee species, Arabica and Robusta, since in the literature it is shown that HCAs are manly present in the Robusta species [5,13,14,15,16,17,18]. Moreover, their identification could also contribute in differentiating the two species, depending on their geographical origin. To our knowledge, HCAs presence in *C. liberica* have not yet been evaluated and their analysis could contribute in the chemical characterization of this species. Caffeoyl-*N*-tryptophan was first isolated in 1987 by Takai et al. [19] from coffee beans of *C. canephora* from Java, while caffeoyl-*N*-tyrosine was first isolated in 1989 by Kellard et al. [20], from *C. canephora* from Angola. Successively, caffeoyl-*N*-phenylalanine, *p*-coumaroyl-*N*-tyrosine, and *p*-coumaroyl-*N*-tryptophan were identified in green coffee beans, analyzing both Robusta and Arabica coffee beans by LC-MS [5,14]. Since these HCAs are not commercially available, their quantification was determined by UV detection using the available standard 5-caffeoylquinic acid (5-CQA) and assuming a similar molar absorption coefficient for 5-CQA and each of the cinnamoyl amides [5]. Usually, chlorogenic acids are the major phenolic compounds present in coffee (30.0–44.4 g/kg) while caffeoyl-*N*-tyrosine and *p*-coumaroyl-*N*-tyrosine were estimated in lower amount (1.3–4.8 g/kg and 1.9–3.7 g/kg, respectively) [5].

HCAs are normally present in plants as enantiomerically enriched compounds, as found, for example, by Hofmann in cocoa beans [11] who isolated *N*-[3′,4′-dihydroxy-(*E*)-cinnamoyl]-L-aspartic acid and verified the absolute configuration of the amino acid by means of polarimetry. Murata et al. [17] isolated from coffee beans *p*-coumaroyl-*N*-tryptophan and demonstrated that L-tryptophan was part of the HCA.

In the present work, 23 samples of green coffee beans (10 of *C. canephora*, 10 of *C. arabica*, and 3 of *C. liberica*) of different geographical origin were analyzed by LC-MS using synthetized *p*-coumaroyl-*N*-tyrosine **1b**, caffeoyl-*N*-phenylalanine **2b**, caffeoyl-*N*-tyrosine **3b**, *p*-coumaroyl-*N*-tryptophan **4b**, and caffeoyl-*N*-tryptophan **5b**. The availability of authentic standards is useful to unequivocally determine their presence in the examined green coffee samples and contribute in the identification of a chiral pool to distinguish the two commercially relevant species.

## 2. Results and Discussion

**Scheme 1:**
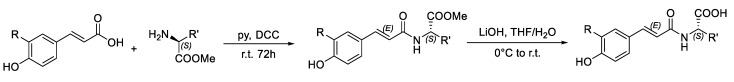
Synthesis of the hydroxycinnamoyl amides.

**Figure 1:**
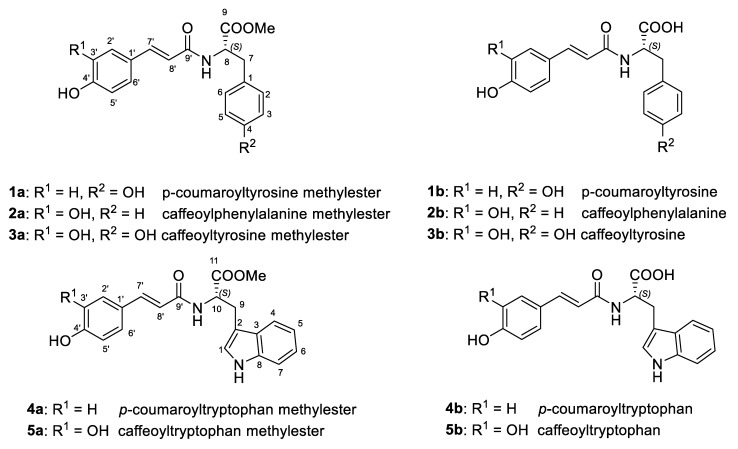
Chemical structures of **1a**–**5a** and **1b**–**5b**.

**Figure 2:**
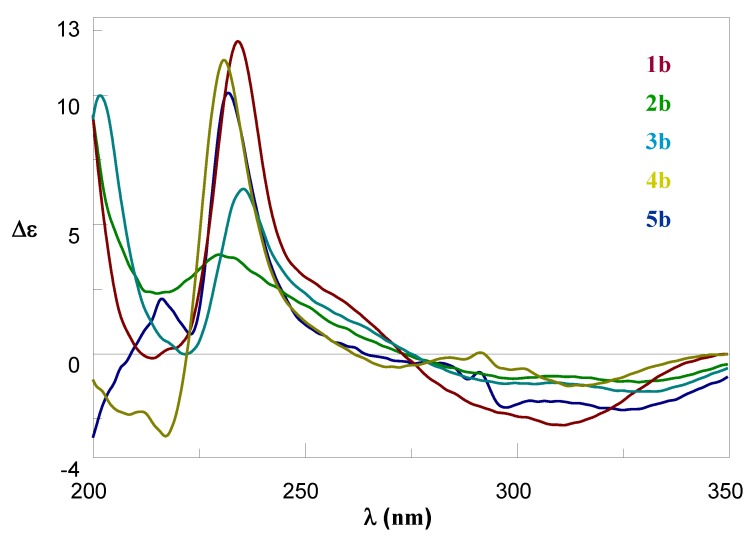
CD spectra of hydroxycinnamoyl amides **1b**–**5b**.

To identify and quantify the HCAs in green coffee beans, the amides **1b**–**5b** were synthetized, which are the compounds of interest in differentiating the two coffee species (Figure 1). Different syntheses of the compounds are reported in the literature [6,9,11], but the best results have been obtained with the procedure of Wang et al. [7], which performed the condensation reaction between the hydroxycinnamic acid and the methyl ester amino acid in pyridine with DCC as the condensing agent. The hydroxycinnamoyl amides methyl esters were obtained from 41% to 78% yield after purification by flash chromatography. These results are in line with the results reported in literature using the same synthetic procedure [7], although for other HCAs. The deprotection reaction with LiOH was a crucial step, and each deprotection was performed at room temperature and was monitored by thin layer chromatography (TLC) in order to determine the end of the reaction, while in the literature, it was performed at 40 °C overnight [7]. In fact, for longer times, we observed a rapid degradation of the products. In this case, better yields were obtained with respect to the literature ranging from 56% to 94%, depending on the purification procedure. For compounds **1b**–**4b** a recrystallization from petroleum ether was performed, while for **5b** it was necessary to purify it by flash chromatography at the expense of a lower yield (56%). All products were amorphous solids. The complete synthesis is reported in Scheme 1. All compounds **1a**–**5a** and **1b**–**5b** were fully characterized by 1D and 2D ^1^H and ^13^C NMR using acetone-d_6_ as the solvent for all the products, while in the literature, different solvents were used (Figure 1). Moreover, chiroptical properties, as optical rotatory power and circular dichroism, were determined for **1b**–**5b**. Circular Dichroism (CD) spectra are reported in Figure 2. Although the isolation of the pure compounds from coffee beans was not the purpose of this work, it was considered important to analyze the chiroptical properties of the synthetized compounds to evaluate, in the future, the correspondence of the chirality of the products with the one isolated from plants.

The synthetized HCAs where analyzed by LC-MS, both in positive and negative mode, to determine their retention time and obtain their mass spectra, in order to unequivocally determine their presence in green coffee bean extract (Table 1).

**Figure 3:**
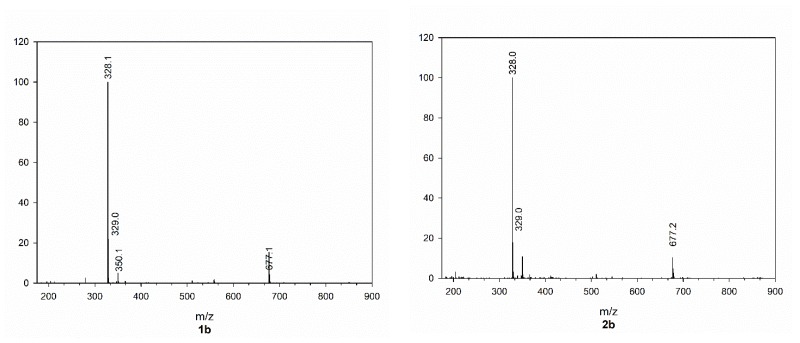

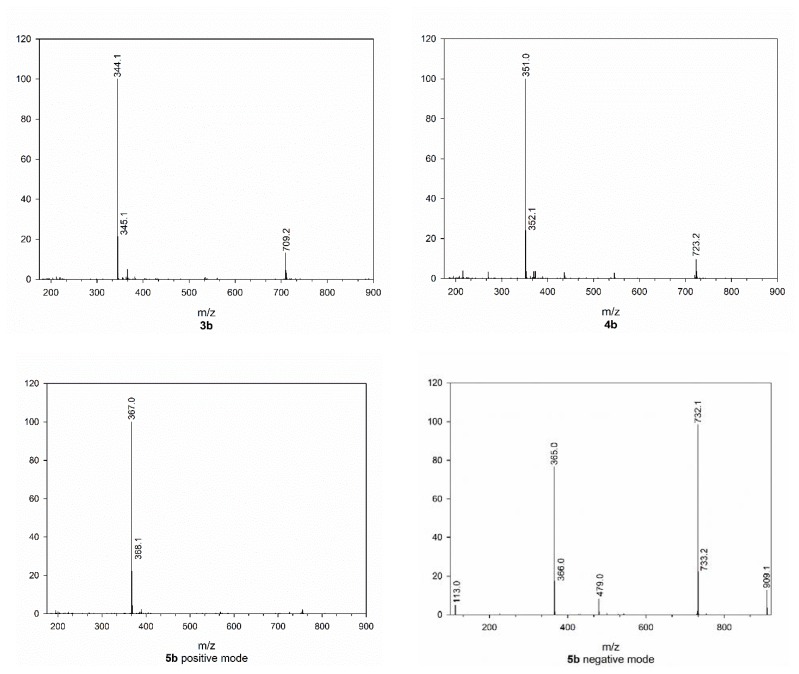
Mass spectra of cinnamoyl amides **1b**–**5b**.

**Figure 4:**
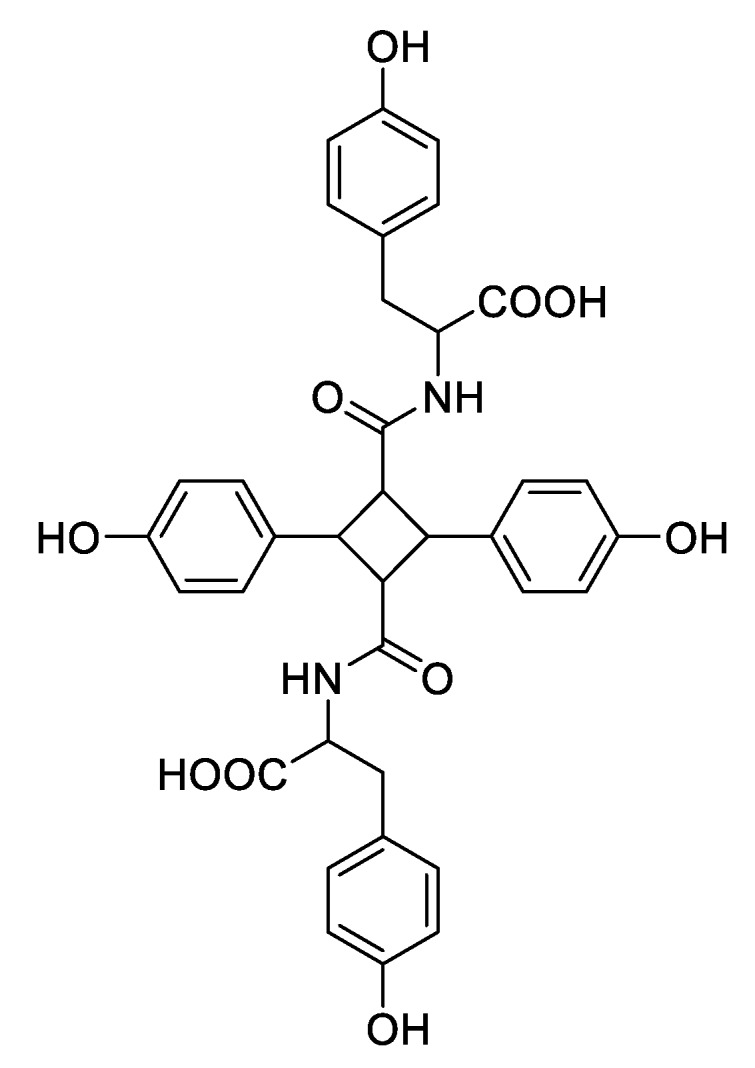
Structure of **1b** dimer.

As can be seen in Figure 3, all mass spectra of HCAs **1b**–**5b** present, besides the quasi molecular ion, an intense peak at higher *m*/*z* ratio, which can be attributed to a dimer that is formed during ionization. The structure of the dimer formed from **1b** is reported in Figure 4 and the assignment of this structure is based on what was found by Yuan et al. in 2014 [8], who isolated these compounds from *Abrus mollis* Hance. Analogues dimers can be expected from the other cinnamoyl amides as confirmed by their *m*/*z* value.

Although only a few works reported the presence of HCAs in coffee beans, it was evidenced in the literature that these compounds were characteristic of the Robusta species [5,13,14,15,16,17,18]. In the present work, compounds **1b**–**5b** have been quantified in green coffee beans of *C. arabica* and *C. canephora* of different geographical origin, and for the first time of *C. liberica*, using an aqueous extraction procedure, as was already used in our previous works for the quantification of chlorogenic acids [21,22].

Quantification of the HCAs **1b** and **4b** was reported to *p*-coumaric acid, and quantification of HCAs **2b**, **3b**, and **5b** was reported to caffeic acid, as suggested by Alonso-Salces [14]. Results are summarized in Table 2 and are given on dry weight basis (DW). A 10% moisture content in green coffee beans was assumed, as it has been done before by others authors [23].

**Figure 5:**
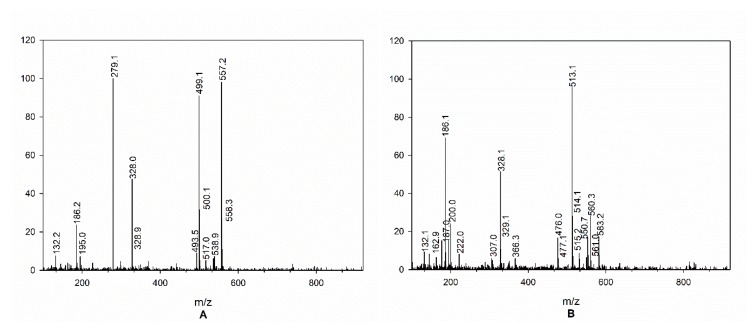
Mass spectra of overlapped peaks of **1b** (**A**) and **2b** (**B**) with unknown compounds.

As can be seen from Table 2, the cinnamoyl amides **1b**–**4b** were present only in the Robusta species, although their presence depended on the geographical origin. Compounds **1b** and **3b** are not present in Robusta green coffee beans from India, Vietnam, and Ivory Coast, while only **3b** is not present in Robusta from Cameroon. Although **1b** and **2b** have been detected in Robusta samples, they could not be quantified, since, in the mass spectra, two other mass peaks were present, indicating that the peak corresponding to the cinnamoyl amides **1b** and **2b** was overlapped with the one of two unknown compounds with mass spectra reported in Figure 5 (positive mode). For this reason, their quantification is not reported in Table 2, and is indicated as not quantifiable (n.q.).

LC-MS analysis of *C. liberica* did not show the presence of any cinnamoyl amide, indicating that it is more similar to the Arabica species than to Robusta, but more samples of Liberica or different extraction methods are needed to verify that HCAs are completely absent in this species.

**Figure 6:**
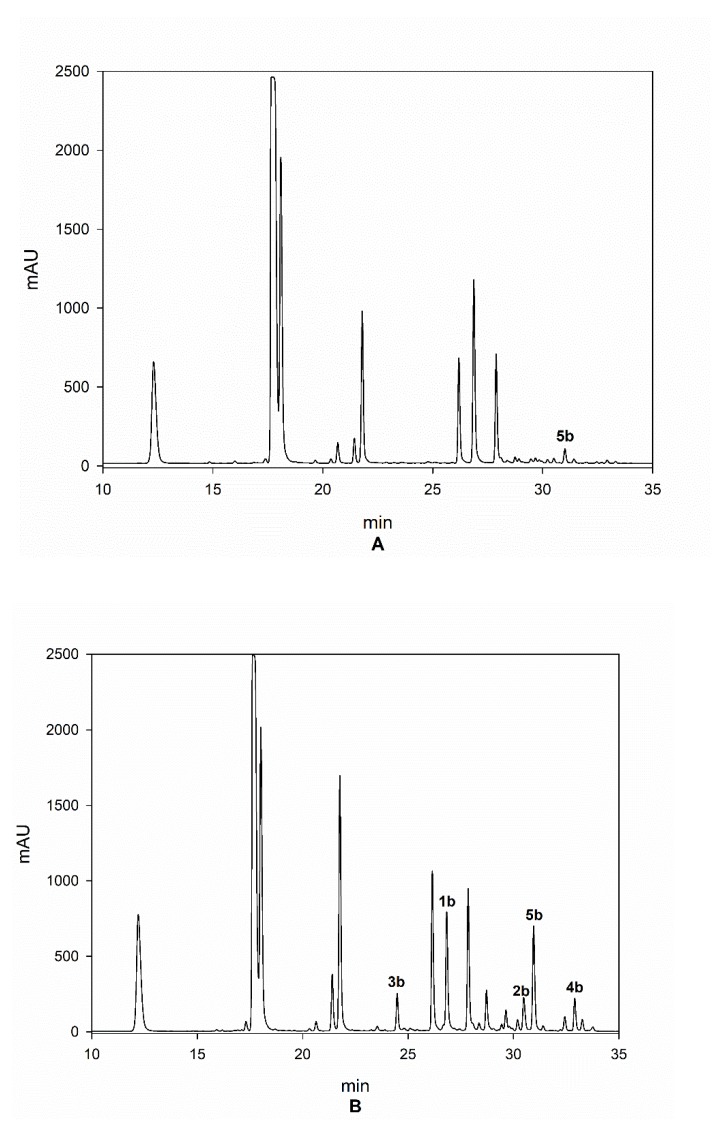

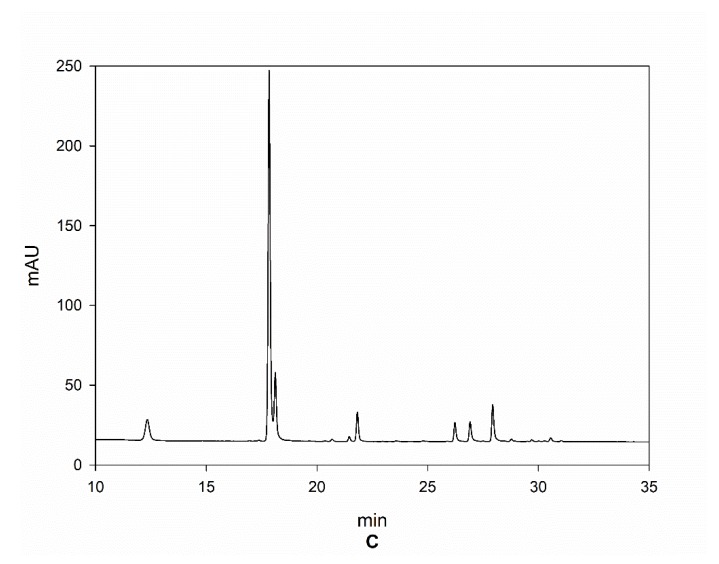
Chromatograms of samples from Arabica India (**A**), Robusta Tanzania (**B**), and Liberica lot 1 (**C**).

In Figure 6, the LC chromatograms referred to the Arabica sample from India, Robusta sample from Tanzania, and Liberica sample—lot 1, are reported as an example, which clearly show the differences between the three species regarding the content of HCAs.

In order to compare the results obtained in the present work with the ones reported in the literature, the extraction method used must be taken into consideration. All methods previously reported, regarding the extraction of HCAs, use an organic solvent, such as methanol [5,17], ethanol [16], or acetone [18], and sometimes, acidic conditions are used [13,14]. Only Rezende et al. [15] and Clifford et al. [24] used hot water for the extraction of HCAs and, in particular, Clifford analyzed the presence of **1b** and **3b**, also in coffee brew. Although the usage of an organic solvent can improve the extraction process, it was interesting to perform the extraction with only hot water to mimic the coffee preparation. The results obtained with this method will be compared in the future with the results obtained in the analysis of coffee brew, to highlight the presence of HCAs also in the final beverage. Nevertheless, results obtained in the present work can be related to the ones reported in the literature by Alonso-Salces [14] and Babova et al. [16] for the two varieties, Arabica and Robusta, although the extraction procedure was different. Moreover, 5-caffeoylquinic acid (5-CQA) is used as standard, assuming that there is a similar molar absorption coefficient for 5-CQA and each of the HCAs [5]; this aspect has to also be taken into consideration when comparing the results with literature data. The results obtained in the present work are in line with the results found by Alonso-Salces [14] since the same compounds for calibration curves were used. The 144 mg/kg of **4b** and 789 of **5b** as a mean value for Robusta samples were obtained, while Alonso-Salces found 225 mg/kg and 1557, respectively. These lower values are probably due to the extraction method, since Alonso-Salces used methanol/water/acetic acid 30:67.5:2.5. The results obtained are also in line with the results found by Babova et al. [16], who obtained 110 mg/kg for **4b** and 400 mg/kg for **5b**, although, using ethanol:water 50:50 as the extraction solvent.

## 3. Materials and Methods

### 3.1. Chemicals

L-tyrosine methyl ester, L-phenylalanine methyl ester, L-tryptophan methyl ester, caffeic acid, *p*-coumaric acid, N,N′-Dicyclohexylcarbodiimide (DCC), LiOH, silica gel were from Sigma-Aldrich (St. Louis, MO, USA). All solvents were used without further purification.

### 3.2. Instrumentation

NMR spectra were recorded on a Varian 400 (Agilent Technologies, Santa Clara, CA, USA) spectrometer using acetone-d_6_ as the solvent. Coupling constants are given in Hz. LC-MS analyses were run on an Agilent Infinity 1200 (Agilent Tech.), with autosampler and a diode array detector set at 320 nm, coupled with an Agilent Technologies 6120 Quadrupole (Agilent Tech.) equipped with an electrospray ionization source (ESI) operating in both positive and negative mode. The column was a Kinetex C18 (150 × 2.1 mm, 100 Å, 5 μm) (Phenomenex, Torrance, CA, USA), and the eluent was a mixture of water + 0.1% formic acid (FA) (*v*/*v*) (solvent A) and acetonitrile + 0.1% FA (*v*/*v*) (solvent B) with the gradient reported in Table 3. The flow was 0.25 mL/min.

Optical rotations were determined on a Jasco P2000 (Jasco, Tokyo, Japan) polarimeter at the wavelength of sodium D (λ = 589 nm) using a quartz cell of 1 dm length. Circular dichroism spectra were recorded on a Jasco J-710 spectropolarimeter using a quartz cell of 0.1 cm optical path.

### 3.3. Samples

A total of 23 samples of green coffee beans from different geographical origins and commercial lots, 10 of *C. arabica*, 10 of *C. canephora*, and 2 of *C. liberica* were analyzed. A wild *C. liberica* (named lot 3) was also used. In particular, all *C. arabica* samples were “wet processed” with zero primary and secondary defects, and the beverages prepared by using the samples were not affected by off-flavors (clean cup). *C. canephora* from India, all “in parchment”, were kindly supplied by Allanasons Private Limited (India), *C. canephora* var. Conilon “dry processed” from Brazil was kindly supplied by Fazendas Dutra (Brazil); all of the other *C*. *canephora* samples were kindly provided by Sandalj Trading Company S.p.A., Trieste (Italy). Two different lots of commercial *C. liberica* “dry processed” from India were kindly provided by Allanasons Private Limited (India), whereas *C. liberica*, named lot 3 from the Costa Rica CATIE germplasm (code T.03476) “in parchment”, was kindly provided by Dr. William Solano Sánchez (Agriculture, Livestock and Agroforestry Program, CATIE, Turrialba, Costa Rica).

### 3.4. General Procedure for the Preparation of the Hydroxycinnamoyl Amides Esters ***1a***–***5a***

The syntheses of all hydroxycinnamoyl amides were performed according to a literature procedure [7].

In a three-necked round bottom flask, 1.58 mmol of the amino acid were dissolved in 12.8 mL of anhydrous pyridine. 2.35 mmol of the hydroxycinnamic acid, and 2.28 mmol of DCC were subsequently added. The reaction mixture was stirred under argon atmosphere for 72 h. At the end of the reaction, the crude was filtered, and the liquid phase was acidified with H_2_SO_4_ 3 M till pH = 2 and washed with a NaHCO_3_ 5% solution. After extraction with ethyl acetate, the organic phase was dried over anhydrous Na_2_SO_4_ and finally evaporated in vacuum. The crude product was purified by silica gel column chromatography using mixtures of petroleum ether/ethyl acetate as eluent.

*N*-[4′-Hydroxy-(*E*)-cinnamoyl]-L-tyrosine methyl ester **1a**: Yield 67%. ^1^H NMR (400 MHz, Acetone-*d*_6_) δ 8.84 (1H, s, OH), 8.27 (1H, s, OH), 7.49 (1H, d, *J* 15.7, H-7′), 7. 47 (1H, NH), 7.43 (2H, d, *J* = 8.6, H-2′, H-6′), 7.06 (2H, d, *J* 8.5, H-2, H-6), 6.86 (2H, d, *J* 8.6, H-3′, H-5′), 6.76 (2H, d, *J* = 8.5, H-3, H-5), 6.62 (1H, d, *J* 15.7, H-8′), 4.81 (1H, dt, *J* 7.7, 5.8, H-8), 3.66 (s, 3H, CH_3_), 3.08 (1H, dd, *J* 13.9, 5.8, H-7), 2.98 (1H, dd, *J* 13.9, 7.7, H-7); ^13^C NMR (100 MHz, Acetone-*d*_6_) δ 172.06 (C-9), 165.8 (C-9′), 159.0 (C-4′), 156.3 (C-4), 140.4 (C-7′), 130.2 (C-2, C-6), 129.5 (C-2′, C-6′), 127.6 (C-1), 126.7 (C-1′), 117.9 (C-8′), 115.7 (C-3′, C-5′), 115.2 (C-3, C-5), 54.1 (C-8), 51.4 (CH_3_), 36.8 (C-7).

*N*-[3′,4′-Dihydroxy-(*E*)-cinnamoyl]-L-phenylalanine methyl ester **2a**: Yield 41%. ^1^H NMR and ^13^C NMR are in accordance with the literature [9].

*N*-Caffeoyl-tyrosine methyl ester **3a**: Yield 58%. ^1^H NMR is in accordance with the literature [9]; ^13^C NMR (100 MHz, Acetone-*d*_6_) δ 172.0 (C-9), 166.0 (C-9′), 156.3 (C-4), 147.2 (C-4′), 145.4 (C-3′), 140.9 (C-7′), 130.2 (C-2 + C-6), 127.5 (C-1), 127.3 (C-1′), 120.9 (C-6′), 117.8 (C-8′), 115.4 (C-5′), 115.2 (C-3 + C-5), 114.1 (C-2′), 54.9 (C-8), 52.2 (COO*C*H_3_), 36.8 (C-7).

*N*-[4′-Hydroxy-(*E*)-cinnamoyl]-L-tryptophan methyl ester **4a**: Yield 72%. ^1^H NMR (400 MHz, Acetone-*d*_6_) δ 10.15 (1H, s, OH), 8.82 (1H, s, NH), 7.65 (1H, d, *J* 7.8, H-4), 7.54 (1H, d, *J* 15.7, H-7′), 7.50 (2H, d, *J* 8.5, H-2′ + H-6′), 7.45 (1H, d, *J* 8.1, H-7), 7.27 (1H, s, H-1), 7.17 (1H, t, *J* 7.9, H-6), 7.09 (1H, t, *J* 7.9, H-5), 6.93 (2H, d, *J* 8.6, H-3′ + H-5′), 6.67 (1H, d, *J* 15.7, H-8′), 5.01 (1H, m, H-10), 3.72 (3H, s, OCH_3_), 3.36 (2H, dd, *J*_1_ 20.0, *J*_2_ 6.4, H-9); ^13^C NMR (100 MHz, Acetone-*d*_6_) δ 173.2 (*C*OOCH_3_), 166.5 (C-9′), 159.8 (C-4′), 141.0 (C-7′), 137.5 (C-8), 130.2 (C-2′ + C-6′), 128.5 (C-1′), 127.6 (C-3), 124.3 (C-1), 122.2 (C-6), 119.6 (C-5), 119.1 (C-4), 118.9 (C-8′), 116.5 (C-3′ + C-5′), 112.2 (C-7), 110.8 (C-2), 54.1 (C-10), 52.2 (COO*C*H_3_), 28.5 (C-9).

*N*-[3′,4′-Dihydroxy-(*E*)-cinnamoyl]-L-tryptophan methyl ester **5a**: Yield 78%. ^1^H NMR (400 MHz, Acetone-*d*_6_) δ 8.33 (1H, s, OH), 8.11 (1H, s, OH), 7.57 (1H, d, *J* 7.6, H-4), 7.50 (1H, NH), 7.45 (1H, d, *J* 15.6, H-7′), 7.37 (1H, d, *J* 8.1, H-7), 7.20 (1H, s, H-1), 7.08 (2H, m, H-2′ + H-5), 7.03 (1H, t, *J* 8.1, H-6), 6.92 (1H, dd, *J*_1_ 8.2, *J*_2_ 2.0, H-6′), 6.82 (1H, d, *J* 8.2, H-5′), 6.56 (1H, d, *J* 15.6, H-8′), 4.93 (1H, m, H-10), 3.65 (3H, s, COOC*H*_3_), 3.30 (2H, ddd, *J*_1_ 21.8, *J*_2_ 14.7, *J*_3_ 6.4, H-9); ^13^C NMR (100 MHz, Acetone-*d*_6_) δ 172.4 (C-11), 165.9 (C-9′), 147.3 (C-7′), 145.4 (C-3′), 140.7 (C-4′), 136.7 (C-8), 127.6 (C-3), 127.6 (C-1′), 123.5 (C-1), 121.3 (C-4), 121.3 (C-6′), 120.9 (C-5), 118.8 (C-6), 118.8 (C-8′), 115.4 (C-5′), 114.0 (C-2′), 111.3 (C-7), 109.9 (C-2), 53.4 (C-10), 51.42 (COO*C*H_3_), 27.6 (C-9).

### 3.5. General Procedure for the Preparation of the Hydroxycinnamoyl Amides ***1b***–***5b***

The 1.11 mmol of the cinnamoylamide ester were dissolved in 68 mL of a THF/H_2_O 1:1 *v*/*v* solution and cooled in an ice bath. Subsequently, 5.53 mmol of LiOH∙H_2_O were added and the mixture was stirred at room temperature. TLC analyses were performed to check the end of the reaction. The mixture was diluted with 50 mL of distilled water and acidified with HCl 3 M till pH 2. After extraction with ethyl acetate, the organic phase was dried over anhydrous Na_2_SO_4_. Evaporation of the solvent under vacuum gave the crude product which was first purified by column chromatography on silica gel using a 99:1 mixture of ethyl acetate: acetic acid as the eluent or by recrystallization from petroleum ether.

(-)-*N*-[4′-Hydroxy-(*E*)-cinnamoyl]-L-tyrosine **1b**: Yield 85%. ^1^H NMR (400 MHz, Acetone-*d*_6_) δ 8.80 (1H, bs, OH), 8.20 (1H, bs, OH), 7.50 (1H, d, *J* 15.7, H-7′), 7.43 (2H, d, *J* 8.6, H-2′, H-6′), 7.31 (1H, d, NH), 7.10 (2H, d, *J* 8.5, H-2, H-6), 6.85 (2H, d, *J* 8.6, H-3′, H-5′), 6.75 (2H, d, *J* 8.5, H-3, H-5), 6.45 (1H, d, *J* 15.7, H-8′), 4.81 (1H, dt, *J* = 7.8, 5.3, H-8), 3.17 (1H, dd, *J* 14.0, 5.3, H-7), 3.00 (1H, dd, *J* 14.0, 7.8, H-7); ^13^C NMR (100 MHz, Acetone-*d*_6_) δ 172.3 (C-9), 165.9 (C-9′), 158.9 (C-4′), 156.1 (C-4), 140.4 (C-7′), 130.3 (C-2, C-6), 129.5 (C-2′, C-6′), 127.8 (C-1), 126.7 (C-1′), 117.9 (C-8′), 115.6 (C-3′, C-5′), 115.1 (C-3, C-5), 53.8 (C-8), 36.5 (C-7); ${\left[\alpha \right]}_{D}^{20}$ −39.9 (*c* 0.1 MeOH) (lit. ${\left[\alpha \right]}_{D}^{20}$ −26.9) [7]; CD (CH_3_OH) Δε_309nm_ = −2.94, Δε_258nm_ = +2.17, Δε_234 nm_ = +13.05; LC-MS (ESI^+^), *m*/*z* 328 ([M + H]^+^), (ESI^−^), *m*/*z* 326 ([M-H]^−^).

(-)-*N*-[3′,4′-Dihydroxy-(*E*)-cinnamoyl]-L-phenylalanine **2b**: Yield 94%. ^1^H NMR (400 MHz, Acetone-*d*_6_) δ 8.30 (OH), 7.44 (1H, d, NH), 7.42 (1H, d, *J* 15.6 Hz, H-7′), 7.35–7.19 (5H, m), 7.08 (1H, d, *J* 2.0 Hz, H-2′), 6.95 (1H, dd, *J* 8.2, 2.0 Hz, H-6′), 6.84 (1H, d, *J* 8.2 Hz, H-5′), 6.56 (1H, d, *J* 15.6 Hz, H-8′), 4.88 (1H, dt, *J* 8.0, 5.4 Hz, H-8), 3.27 (1H, dd, *J* 13.9, 5.4 Hz, H-7), 3.09 (1H, dd, *J* 13.9, 8.0 Hz, H-7); ^13^C NMR (100 MHz, Acetone-*d*_6_) δ 172.2 (C-9), 165.7 (C-9′), 147.0 (, 145.2, 140.6 (H-7′), 137.4 (Ph), 129.3 (Ph), 128.3 (Ph), 127.4 (Ph), 126.6 (Ph), 120.9 (C-6′), 118.0 (C-8′), 115.4 (C-5′), 114.0 (C-2′), 53.5 (C-8), 37.3 (C-7); ${\left[\alpha \right]}_{D}^{20}$ −48.8 (*c* 0.1, MeOH); CD (MeOH) Δε_330nm_ = −1.1, Δε_297nm_ = −1.0, Δε_250nm_ = +1.8, Δε_230nm_ = +3.9; LC-MS (ESI^+^), *m*/*z* 328 ([M + H]^+^), (ESI^−^), *m*/*z* 326 ([M-H]^−^).

(-)-*N*-[3′,4′-Dihydroxy-(*E*)-cinnamoyl]-L-tyrosine **3b**: yield 84%. ^1^H NMR (400 MHz, Acetone-*d*_6_) δ 7.36 (1H, d, *J* 15.6, H-7′), 7.26 (1H, NH), 7.05 (2H, d, *J* 8.6, H-2 + H-6), 7.03 (1H, d, *J* 2.0, H-2′), 6.90 (1H, dd, *J*_1_ 8.2, *J*_2_ 2.0, H-6′), 6.78 (1H, d, *J* 8.2 Hz, H-5′), 6.70 (2H, d, *J* 8.6, H-3 + H-5), 6.51 (1H, d, *J* 15.6, H-8′), 4.75 (1H, dt, *J*_1_ 7.8, *J*_2_ 5.3, H-8), 3.10 (1H, dd, *J*_1_ 14.0, *J*_2_ 5.3, H-7), 2.95 (1H, dd, *J*_1_ 14.0, *J*_2_ 7.8, H-7); ^13^C NMR (100 MHz, Acetone-*d*_6_) δ 173.2 (C-9), 166.6 (C-9′), 157.0 (C-4), 147.9 (C-4′), 146.1 (C-3′), 141.4 (C-7′), 131.1 (C-2 + C-6), 128.7 (C-1), 128.3 (C-1′), 121.7 (C-6′), 119.0 (C-8′), 116.3 (C-5′), 115.9 (C-3 + C-5), 114.9 (C-2′), 54.7 (C-8), 37.7 (C-7); ${\left[\alpha \right]}_{D}^{25}$ −41.7 (*c* 0.1, MeOH) (lit. ${\left[\alpha \right]}_{D}^{20}$ −35.6) [11]; CD (MeOH): Δε_331_ = −1.5, Δε_269_ = +0.4, Δε_235_ = +6.5, Δε_221_ = −0.2; LC-MS (ESI^+^), *m*/*z* 344.1 ([M + H]^+^), (ESI^−^), *m*/*z* 342.0 ([M-H]^−^).

(-)-*N*-[4′-Hydroxy-(*E*)-cinnamoyl]-L-tryptophan **4b**: yield 91%. ^1^H NMR (400 MHz, Acetone-*d*_6_) δ 10.05 (1H, s, OH), 8.90 (1H, bs, COOH), 7.61 (1H, d, *J* 7.9, H-4), 7.51 (2H, m, H-7′+NH), 7.37 (2H, d, *J* 8.6, H-6′ + H-2′), 7.33 (1H, d, *J* 8.1, H-7), 7.20 (1H, s, H-1), 7.04 (1H, t, *J* 7.9, H-6), 6.96 (1H, t, *J* 7.9, H-5), 6.82 (2H, d, *J* 8.6, H-5′ + H-3′), 6.60 (1H, d, *J* 15.7, H-8′), 4.99 (1H, m, H-10), 3.42 (1H, dd, *J*_1_ 22.2, *J*_2_ 14.8, H-9), 3.28 (1H, dd, *J*_1_ 22.2, *J*_2_ 6.3, H-9); ^13^C NMR (100 MHz, Acetone-*d*_6_) δ 172.9 (COOH), 166.4 (C-9′), 159.2 (C-4′), 140.6 (C-7′), 136.7 (C-8), 129.6 (C-2′ + C-6′), 127.9 (C-1′), 126.7 (C-3), 123.6 (C-1), 121.4 (C-6), 118.8 (C-5), 118.5 (C-4), 117.9 (C-8′), 115.8 (C-3′ + C-5′), 111.4 (C-7), 110.1 (C-2), 53.4 (C-10), 27.6 (C-9); ${\left[\alpha \right]}_{D}^{25}$ −27.8 (*c* 0.1, MeOH); CD (MeOH): Δε_323_ = −1.4, Δε_292_ = −0.1, Δε_270_ = −0.5, Δε_250_ = +1.2, Δε_231_ = +11.7, Δε_217_ = −3.5; LC-MS (ESI^+^), *m*/*z* 351.0 ([M + H]^+^), (ESI^−^), *m*/*z* 349.1 ([M-H]^−^).

(-)-*N*-[3′,4′-Dihydroxy-(*E*)-cinnamoyl]-L-tryptophan **5b**: Yield 56%. ^1^H NMR (400 MHz, Acetone-*d*_6_) δ 10.06 (1H, s, OH), 7.59 (1H, d, *J* 7.6, H-4), 7.40 (1H, d, *J* 15.6, H-7′), 7.36 (1H, NH), 7.32 (1H, d, *J* 8.2, H-7), 7.17 (1H, d, *J* 2.0, H-1), 7.02 (2H, m, H-5 + H-2′), 6.96 (1H, t, *J* 7.6, H-6), 6.88 (1H, dd, J_1_ 8.2, J_2_ 2.0, H-6′), 6.77 (1H, d, *J* 8.2, H-5′), 6.53 (1H, d, *J* 15.6, H-8′), 4.94 (1H, m, H-10), 3.37 (1H, dd, *J*_1_ 14.8, *J*_2_ 5.3, H-9), 3.24 (1H, dd, *J*_1_14.8, *J*_2_ 7.3, H-9); ^13^C NMR (100 MHz, Acetone-*d*_6_) δ 173.5 (C-11), 166.8 (C-9′), 147.9 (C-7′), 146.1 (C-3′), 141.4 (C-4′), 137.5 (C-8), 128.7 (C-3), 128.3 (C-1′), 124.3 (C-1), 122.1 (C-4), 121.7 (C-6′), 119.5 (C-5), 119.3 (C-6), 119.0 (C-8′), 116.2 (C-5′), 114.9 (C-2′), 112.1 (C-7), 111.0 (C-2), 54.0 (C-10), 28.3 (C-9). ${\left[\alpha \right]}_{D}^{25}\;$−33.2 (*c* 1.0, MeOH); CD (MeOH): Δε_325_ = −2.3, Δε_298_ = −2.1, Δε_232_ = +10.2, Δε_216_ = +2.4; LC-MS (ESI^+^), *m*/*z* 367.1 ([M + H]^+^), (ESI^−^), *m*/*z* 365.1 ([M-H]^−^).

### 3.6. Extraction of Hydroxycinnamoyl Amides and Sample Preparation

Green coffee beans were ground to a fine powder in a mixer ball mill MM400 (Retsch, Haan, Germany) and extraction was performed in duplicate by dynamic heat-assisted water extraction [15,16]. For this purpose, 1 g of powdered green coffee from each species was added to 100 mL of boiling water and the mixture was stirred for 10 min at 200 rpm on a heated plate (Arex Velp Scientifica, Usmate, Italy) and filtered. The aqueous extract was frozen with liquid nitrogen and freeze dried for 3 days.

HPLC samples were prepared dissolving the lyophilized extracts in MilliQ water (obtained with a Milli-Q Reference Water Purification System, Merck, Darmstadt, Germany) at a concentration of 6 mg/mL. Subsequently the solutions were filtered with 0.2 μm PTFE syringe filters (from VWR International Ltd., Radnor, PA, USA), preconditioned with methanol and injected to LC-MS.

### 3.7. Quantitative Analyses

To quantify the hydroxycinnamoyl amides, caffeic acid and *p*-coumaric acid were used as standard compounds, exploiting their UV absorption at 320 nm for detection, as already reported in the literature [14]. Calibration curves of caffeic acid and p-coumaric acid were built reporting the integration signal (mAu*s) versus concentration (μg/mL) as mean value of triplicate analyses in the range of concentration 0.035–3.5 μg/mL and 0.041–4.1 μg/mL, respectively. Both calibration curves showed a good response linearity with a coefficient of correlation (R^2^) of 0.997. Limits of detection (LOD) and quantification (LOQ) were, respectively, 0.14 μg/mL and 0.20 μg/mL calculated as signal-to-noise ratio with S:N = 3 for LOD and S:N = 10 for LOQ.

## 4. Conclusions

The results obtained in the present work are in accordance with the literature demonstrating that the HCAs *p*-coumaroyl-*N*-tyrosine **1b**, caffeoyl-*N*-phenylalanine **2b**, caffeoyl-*N*-tyrosine **3b**, and *p*-coumaroyl-*N*-tryptophan **4b**, and caffeoyl-*N*-tryptophan **5b**, are characteristic of the Robusta green coffee beans, although their distribution between the samples depends on the geographical origin. Samples from Tanzania and Uganda contain all five HCAs while Robusta from Indonesia has only *p*-coumaroyl-*N*-tryptophan **4b** and caffeoyl-*N*-tryptophan **5b**. The only HCA present in Arabica is caffeoyl-*N*-tryptophan **5b**, although in a very small amount with respect to Robusta. Liberica didn’t show the presence of any HCAs, although a small number of samples were considered. The investigation on more samples of Liberica of different origins is necessary to confirm the absence of HCAs. Preliminary studies on roasted coffee beans of both Robusta and Arabica were carried out by Kim et al. [13], where it was evidenced that the HCAs content decreased. A future development of the HCAs analysis could consider coffee brews from different commercial sources to evaluate if the HCAs content can discriminate on the coffee species used.

## Figures and Tables

**Table 1:** HPLC retention times, MS analysis, and specific rotation.

Compound	t_R_ (min)	[M + H] (*m*/*z*) Exp.	Dimer + Na (*m*/*z*) Exp.	Specific Rotation
*p*-coumaroyl-*N*-tyrosine **1b**	26.9	328.1	677.1 (positive mode)	${\left[\alpha \right]}_{D}^{20}$ −39.9 (*c* 0.1 MeOH) (lit. ${\left[\alpha \right]}_{D}^{20}$ −26.9 (MeOH) [11] and ${\left[\alpha \right]}_{D}^{20}$ −17.7 (*c* 0.1 MeOH) [7])
caffeoyl-*N*-phenylalanine **2b**	30.6	328.0	677.2 (positive mode)	${\left[\alpha \right]}_{D}^{20}$ −48.8 (*c* 0.1 MeOH)
caffeoyl-*N*-tyrosine **3b**	24.6	344.1	709.2 (positive mode)	${\left[\alpha \right]}_{D}^{25}$ −41.7 (*c* 0.1, MeOH) (lit. ${\left[\alpha \right]}_{D}^{20}$ −35.6 [11])
*p*-coumaroyl-*N*-tryptophan **4b**	33.0	351.0	723.2 (positive mode)	${\left[\alpha \right]}_{D}^{25}$ −27.8 (*c* 0.1, MeOH)
caffeoyl-*N*-tryptophan **5b**	31.1	367.1	731.1 (negative mode)	${\left[\alpha \right]}_{D}^{25}$ −33.2 (*c* 1.0, MeOH)

**Table 2:** Concentrations of cinnamoyl amides in green coffee beans (mg/kg of DW, standard deviation in brackets).

Sample	Species	1b	2b	3b	4b	5b	Total
1	*C. arabica* Nicaragua	<LOD	<LOD	<LOD	<LOD	117 (2)	117 (2)
2	*C. arabica* Peru	<LOD	<LOD	<LOD	<LOD	<LOD	-
3	*C. arabica* Rwanda	<LOD	<LOD	<LOD	<LOD	51 (1)	51 (1)
4	*C. arabica* El Salvador	<LOD	<LOD	<LOD	<LOD	<LOD	-
5	*C. arabica* India	<LOD	<LOD	<LOD	<LOD	125 (3)	125 (3)
6	*C. arabica* Brazil	<LOD	<LOD	<LOD	<LOD	37 (1)	37 (1)
7	*C. arabica* Guatemala	<LOD	<LOD	<LOD	<LOD	97 (2)	97 (2)
8	*C. arabica* – lot 1 Ethiopia	<LOD	<LOD	<LOD	<LOD	59 (1)	59 (1)
9	*C. arabica* – lot 2 Ethiopia	<LOD	<LOD	<LOD	<LOD	75 (2)	75 (2)
10	*C. arabica* Burundi	<LOD	<LOD	<LOD	<LOD	40 (1)	40 (1)
11	*C. canephora* Cameroon	n.q. ^a^	n.q. ^b^	<LOD	119 (4)	878 (26)	997 (30)
12	*C. canephora* Ivory Coast	<LOD	n.q. ^b^	<LOD	116 (4)	712 (21)	828 (25)
13	*C. canephora* Tanzania	n.q. ^a^	n.q. ^b^	348 (5)	319 (3)	1042 (14)	1709 (22)
14	*C. canephora* Indonesia	<LOD	<LOD	<LOD	113 (2)	590 (12)	703 (14)
15	*C. canephora* – lot 1 India	<LOD	n.q. ^b^	<LOD	103 (3)	790 (24)	893 (27)
16	*C. canephora* – lot 2 India	<LOD	n.q. ^b^	<LOD	212 (6)	1205 (48)	1417 (54)
17	*C. canephora* – lot 3 India	<LOD	n.q. ^b^	<LOD	128 (4)	780 (23)	908 (27)
18	*C. canephora* Brazil var. Conilon	n.q. ^a^	47 (1)	182 (4)	<LOD	470 (9)	699 (14)
19	*C. canephora* Uganda	n.q. ^a^	n.q. ^b^	140 (3)	195 (5)	717 (21)	1052 (29)
20	*C. canephora* Vietnam	<LOD	n.q. ^b^	<LOD	136 (3)	702 (20)	838 (23)
21	*C. liberica* – lot 1	<LOD	<LOD	<LOD	<LOD	<LOD	-
22	*C. liberica* – lot 2	<LOD	<LOD	<LOD	<LOD	<LOD	-
23	*C. liberica* – lot 3	<LOD	<LOD	<LOD	<LOD	<LOD	-

n.q.: not quantifiable: ^a^ overlapped with unknown compound with *m*/*z* 557, 499, and 279; ^b^ overlapped with unknown compound with *m*/*z* 513 and 186; DW: dry weight; LOD: limit of detection.

**Table 3:** HPLC gradient method.

% A (H_2_O + 0.1% FA)	% B (CH_3_CN + 0.1% FA)	Time (min)
97	3	0
85	15	10
60	40	30
60	40	40
97	3	45

## Abbreviations

CD	Circular Dichroism
5-CQA	5-caffeoylquinic acid
DCC	N,N′-Dicyclohexylcarbodiimide
DW	Dry weight
FA	Formic acid
HCA	Hydroxycinnamoyl amide
